# Effect of replacing television viewing with different intensities of physical activity on COVID-19 mortality risk: Short communication from UK Biobank

**DOI:** 10.1177/14034948231158441

**Published:** 2023-03-08

**Authors:** Malik Hamrouni, Nicolette Bishop

**Affiliations:** National Centre for Sport and Exercise Medicine, School of Sport, Exercise and Health Sciences, Loughborough University, UK

**Keywords:** Sedentary behaviour, inactivity, screen-time, coronavirus, epidemiology

## Abstract

**Aims:**

This study aimed to examine the theoretical effects of replacing television (TV) viewing with different intensities of physical activity on COVID-19 mortality risk using isotemporal substitution models.

**Methods:**

The analytical sample was composed of 359,756 UK Biobank participants. TV viewing and physical activity were assessed by self-report. Logistic regressions adjusted for covariates were used to model the effects of substituting an hour a day of TV viewing with an hour of walking, moderate-intensity physical activity (MPA) or vigorous-intensity physical activity (VPA) on COVID-19 mortality risk.

**Results:**

From 16 March 2020 to 12 November 2021, there were 879 COVID-19 deaths in the analytical sample. Substituting an hour a day of TV viewing with an hour of walking was associated with a 17% lower risk of COVID-19 mortality (odds ratio (OR)=0.83, 95% confidence interval (CI) 0.74–0.92). In sex-stratified analyses, the same substitution was associated with a lower risk in both men (OR=0.85, 95% CI 0.74–0.96) and women (OR=0.78, 95% CI 0.65–0.95). However, replacing an hour a day of TV viewing with an hour of MPA was only associated with a lower risk in women (OR=0.80, 95% CI 0.65–0.98).

**Conclusions:**

**Replacing TV viewing with walking was associated with a significant reduction in COVID-19 mortality risk. Public health authorities should consider promoting the replacement of TV viewing with walking as a protective strategy against COVID-19 mortality.**

## Introduction

Physical inactivity and a high television (TV) viewing time have been shown to be risk factors for severe disease and mortality from COVID-19 [1–3]. Whilst the available research suggests that increasing physical activity and reducing TV viewing time may confer a protective effect against COVID-19, studies so far have only demonstrated the effect of a particular behaviour without considering that a change in said behaviour may displace other behaviours [4].

Isotemporal substitution models allow for the assessment of the theoretical effect on health outcomes of replacing one behaviour with another in an equal time-exchange manner [5]. As highlighted by Mekary et al., this accounts for the confounding effect of other relevant behaviours when assessing the role of a specific behaviour and enables better application of findings to public health recommendations [4].

Therefore, using data provided by UK Biobank, isotemporal substitution models were used to explore the theoretical effect of replacing TV viewing with walking, moderate-intensity physical activity (MPA) and vigorous-intensity physical activity (VPA) on the risk of COVID-19 mortality.

## Methods

All data were provided by UK Biobank, a cohort of >500,000 individuals recruited from the general population, aged 37–73 years at the time of baseline assessment (2006–2010) [6]. Exposure variables and covariates were assessed at baseline. COVID-19 mortality data were obtained via linkage of the cohort with NHS Digital. The analytical period for this study was 16 March 2020–12 November 2021. UK Biobank obtained ethical approval from the North West Multi-centre Research Ethics Committee. All participants gave written informed consent.

Baseline age was calculated from date of birth, and NHS records were used to determine sex. Height and weight were used to calculate body mass index (BMI; kg/m^2^). A touchscreen questionnaire was completed by participants to determine ethnicity, tobacco smoking and alcohol intake frequency, and number of cancer and non-cancer illnesses. The Townsend Index was used as a proxy for material deprivation and was determined from each participant’s postcode at recruitment. The Townsend Index is an area-level deprivation metric calculated based on non-home ownership, non-car ownership, unemployment and overcrowding within any geographical area [7]. The Townsend Index has been shown to be strongly correlated with measures of deprivation calculated at the individual level and to be similarly predictive of health [8]. Self-reported physical activity (for walking, MPA and VPA) was assessed using questions adapted from the International Physical Activity Questionnaire (IPAQ) short form [9]. The questions captured the frequency (days per week) and duration (minutes per day) of walking, MPA and VPA on a typical day. This information was then used to calculate hours per day spent doing each behaviour. All physical activity data processing was carried out in line with IPAQ guidelines [10]. To obtain TV viewing time, participants were asked how many hours they spend on a typical day watching TV. Values between 16 and 24 hours per day were recoded as 16 hours per day to minimise the influence of possible spurious outliers (i.e. winsorizing), in line with previous UK Biobank studies [11,12]. Mortality from COVID-19 was defined as the presence of ICD-10 code U071 (virus identified in laboratory testing) or U072 (clinical or epidemiological diagnosis) as a primary or contributary cause on the death certificate.

Isotemporal substitution models were used to examine the effect of replacing an hour a day of TV viewing with an hour of walking, MPA or VPA on COVID-19 mortality risk. These models included walking time, MPA time, VPA time and the total time in all behaviours (i.e. walking+MPA+VPA+TV viewing) [13]. By removing TV viewing time from these models and including total time in all behaviours, the output from these models represents the effect of replacing an hour a day of TV viewing with the same amount of time of another behaviour [13]. The aforementioned analyses were conducted across the whole sample and then stratified by sex. Performing sex-stratified analyses was determined a priori based on previous research suggesting there may be sex-based differences in the protective association of physical activity against COVID-19 [14]. However, we also tested whether there were significant interaction effects of sex with our exposure variables. Potential confounders included baseline age, sex, ethnicity, tobacco smoking and alcohol intake frequency; Townsend Index; and number of cancer and non-cancer illnesses. In line with Rowlands et al., BMI was not adjusted for in the main analysis, as it may be on the causal pathway for the protective effect of physical activity against COVID-19 [15]. However, a sensitivity analysis was performed whereby all models were further adjusted for BMI. Results are reported as odds ratios (ORs) with 95% confidence intervals (CI). Statistical significance was accepted at *p*<0.05. All statistical analyses were performed using R (The R Foundation for Statistical Computing, R version 4.2.0, Vienna, Austria).

## Results

**Figure 1.**
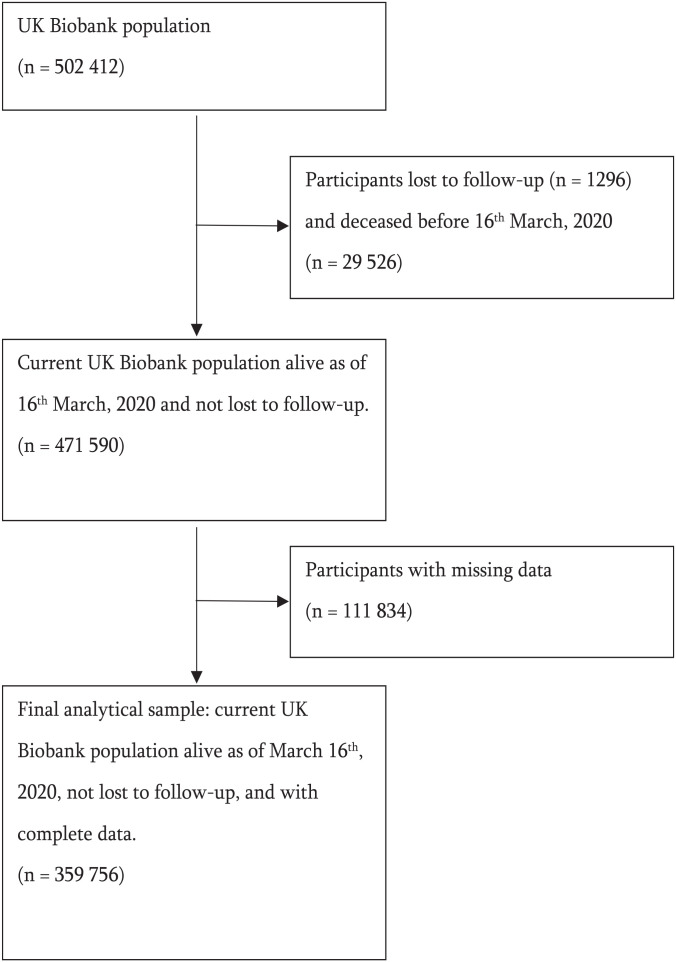
Participant flow diagram.

The final analytical sample was composed of 359,756 individuals after removing those who were lost to follow-up, those who died before 16 March 2020 and those with missing data. There were 879 COVID-19 deaths over the analytical period (16 March 2020 to 12 November 2021). Participant demographics for the full sample and stratified by sex are presented in the Table I. A participant flow diagram is shown in Figure 1. The number of participants with missing data for each variable can be found in the Supplemental Material (Supplemental Table SI). A notable number of participants had missing data for either walking, MPA or VPA (23%). Participant demographics for those who did and did not die from COVID-19 are presented in the Supplemental Material (Supplemental Table SII).

**Table I.** Participant demographics for the full sample and stratified by sex.

Variable	Full sample (*N*=359,756)	Men (*N*=169,423)	Women (*N*=190,333)
Number of COVID-19 deaths	879 (<1%)	596 (<1%)	283 (<1%)
Baseline age (years)	56 (8)	56 (8)	56 (8)
Current age (years)^a^	67 (8)	68 (8)	67 (8)
BMI (kg/m^2^)	27.2 (4.6)	27.7 (4.1)	26.8 (5.0)
Townsend Index^b^	−1.5 (3.0)	−1.5 (3.0)	−1.5 (3.0)
Number of cancer and noncancer illnesses	2 (2)	2 (2)	2 (2)
TV viewing (h/day)	2.64 (1.62)	2.68 (1.64)	2.61 (1.60)
Walking (h/day)	0.76 (0.79)	0.76 (0.80)	0.75 (0.77)
MPA (h/day)	0.58 (0.75)	0.58 (0.75)	0.55 (0.71)
VPA (h/day)	0.21 (0.36)	0.25 (0.41)	0.18 (0.30)
Ethnicity			
White	342,164 (95%)	161,272 (95%)	180,892 (95%)
Non-white	17,592 (5%)	8151 (5%)	9441 (5%)
Tobacco smoking frequency			
On most or all days	24,503 (7%)	12,969 (8%)	11,534 (6%)
Only occasionally	9867 (3%)	5854 (3%)	4013 (2%)
Non-smoker	325,386 (90%)	150,600 (89%)	174,786 (92%)
Alcohol intake frequency			
Daily/almost daily	76,693 (21%)	44,216 (26%)	32,477 (17%)
1–4 times a week	181,085 (50%)	89,807 (53%)	91,278 (48%)
1–3 times a month	39,727 (11%)	14,906 (9%)	24,821 (13%)
Special occasions/never	62,251 (17%)	20,494 (12%)	41,757 (22%)

Characteristics presented as mean (standard deviation) for continuous variables and as number (percentage) for categorical variables.

a Age as of 1 March 2020.

b Higher values for the Townsend Index imply a greater degree of deprivation.

BMI: body mass index; TV: television; MPA: moderate-intensity physical activity; VPA: vigorous-intensity physical activity.

The isotemporal substitution models are shown in Table II. For the full sample, the isotemporal substitution models demonstrated that replacing an hour a day of TV viewing with an hour of walking was associated with a significantly lower risk of COVID-19 mortality. Sex-stratified analyses were performed, although no statistically significant interactions for sex with our exposure variables were observed. In both men and women, replacing an hour a day of TV viewing with an hour of walking was associated with a significantly lower risk of COVID-19 mortality. However, replacing an hour a day of TV viewing with an hour of MPA was only associated with a significantly lower risk in women.

**Table II.** Isotemporal substitution models examining the effect of replacing TV viewing with walking, MPA and VPA on COVID-19 mortality risk.

	Walking	MPA	VPA
Full sample	**0.83 (0.74–0.92)**	0.90 (0.81–1.01)	0.86 (0.70–1.06)
Men	**0.85 (0.74–0.96)**	0.96 (0.84–1.09)	0.83 (0.65–1.05)
Women	**0.78 (0.65–0.95)**	**0.80 (0.65–0.98)**	0.94 (0.62–1.42)

Odds ratios (95% confidence intervals) for COVID-19 mortality when replacing an hour of TV viewing with an hour of each physical activity. All physical behaviours except for TV viewing were entered into the model with adjustment for total time in all behaviours (i.e. TV viewing+walking+MPA+VPA). All models were adjusted for baseline age, sex (except when performing sex-specific analyses), ethnicity, tobacco smoking and alcohol intake frequency, Townsend Index and number of cancer and noncancer illnesses. Significant associations (*p*<0.05) are shown in bold.

Including BMI into the regression models rendered several associations no longer significant (Supplemental Table SIII). However, replacing an hour a day of TV viewing with walking was still associated with a significantly lower risk of COVID-19 mortality when analysing the full sample.

## Discussion

The isotemporal substitution models used in this study are the first to model the effect of replacing an hour a day of TV viewing with different physical activities (walking, MPA and VPA) on COVID-19 mortality risk. Across the full sample and the sex-stratified analyses, substituting an hour a day of TV viewing with walking was associated with a lower risk of COVID-19 mortality. However, replacing an hour a day of TV viewing with MPA was only associated with a lower risk in women.

When analysing the full sample, differential changes in COVID-19 mortality risk were found when replacing an hour a day of TV viewing with walking compared to MPA and VPA. The stronger associations observed for walking compared to MPA and VPA – which persisted even after adjustment for BMI in the sensitivity analysis – are interesting, given that some studies suggest that higher-intensity physical activity may confer greater health benefits [16,17]. However, this is not always the case. Using isotemporal substitution models to assess how replacing TV viewing with walking, MPA and VPA may modify the risk of developing specific cancers, Hunter et al. observed several instances in which substituting with walking but not MPA or VPA was associated with a lower risk (e.g. lung cancer) [13]. Similarly, Stamatakis et al. found that in individuals with a high sitting time (>6 hours a day) replacing an hour a day of sitting with walking but not MPA was associated with a lower risk of all-cause mortality [18].

Although there were no statistically significant interaction effects for sex, replacing an hour a day of TV viewing with MPA was associated with a significantly lower risk of COVID-19 mortality in women but not in men. The potentially stronger protective association of physical activity in women observed herein is consistent with previous findings from Rowlands et al., who found stronger relationships for all of the analysed physical activity metrics in women compared to men in relation to risk of severe COVID-19 [14]. Given that men are at disproportionately higher risk of severe COVID-19 [19], the ability for modifiable lifestyle factors such as physical activity to mitigate risk may be reduced. The current findings highlight the importance of not solely including sex as a confounder in studies examining the role of physical activity, as doing so may mask potential sex-based differences regarding the protective associations of physical activity.

The current study has several limitations. The analysed physical behaviours were measured by self-report, making them subject to recall and social desirability bias. The progression through walking to MPA to VPA reflects increases in activity intensity. However, the intensities within each activity domain are not necessarily homogenous. Indeed, research has shown that brisk walking may confer greater health benefits than slower walking [20,21]. Future studies using isotemporal substitution with stratification by time spent at different walking intensities (as done by Mekary et al. [4]) may help to elucidate further the protective association of walking against COVID-19. Furthermore, the UK Biobank population is healthier than the general population [22], meaning that the analysed sample is not representative. However, this likely does not affect the identification of risk factors for diseases [23]. As this is an observational study, causal associations cannot be inferred from the results, and the potential for confounding from unmeasured covariates cannot be dismissed. Although we adjusted for the Townsend Index (an area-level deprivation metric which has been shown to be strongly correlated with individual-level deprivation metrics) [8], future research may nevertheless benefit from further adjustment for individual-level data (e.g. occupation/employment status, income and education), in addition to exploring/comparing the extent to which area- and individual-level deprivation may modify the effect of TV viewing and physical activity on COVID-19 mortality risk. Another limitation of this study is that we observed a high number of participants with missing physical activity data, which may reduce statistical power, introduce selection bias and reduce the representativeness of the sample [24]. When interpreting the findings, one should also consider that a much greater number of participants did not partake in any MPA or VPA compared to walking (14% and 37% of the analytical sample did not perform any MPA and VPA, respectively, compared to 2% not performing any walking). This may have hindered our ability to detect significant protective associations for replacing TV viewing with MPA and VPA and may explain the markedly wider confidence intervals observed for VPA compared to walking.

In conclusion, the key finding of this short communication was that replacing TV viewing with walking was associated with a significant reduction in risk of COVID-19 mortality. Although future research investigating mechanisms is required to determine the biological plausibility of the present findings, the results are encouraging, especially considering that walking is a more convenient physical activity to partake in compared to MPA and VPA for most individuals. Based on the current results, public health authorities should consider promoting the replacement of TV viewing with walking as a protective strategy against COVID-19 mortality.

## Supplemental Material

sj-docx-1-sjp-10.1177_14034948231158441 – Supplemental material for Effect of replacing television viewing with different intensities of physical activity on COVID-19 mortality risk: Short communication from UK Biobank Click here for additional data file. Supplemental material, sj-docx-1-sjp-10.1177_14034948231158441 for Effect of replacing television viewing with different intensities of physical activity on COVID-19 mortality risk: Short communication from UK Biobank by Malik Hamrouni and Nicolette Bishop in Scandinavian Journal of Public Health
