# Supplementary material for: Effect of replacing television viewing with different intensities of physical activity on COVID-19 mortality risk: Short communication from UK Biobank
Source: Scand J Public Health. 2023 Mar 8;51(5):786–91. doi: 10.1177/14034948231158441 (PMC9996135; doi:10.1177/14034948231158441)
Supplement: sj-docx-1-sjp-10.1177_14034948231158441 – Supplemental material for Effect of replacing television viewing with different intensities of physical activity on COVID-19 mortality risk: Short communication from UK Biobank [file sj-docx-1-sjp-10.1177_14034948231158441.docx]

| **Supplementary Table 1.** Variables with missing data for the 471 590 UK Biobank participants alive as of March 16^th^ 2020 and not lost to follow up. | |
| --- | --- |
| **Variable** | **N missing (%)** |
| **Continuous variables** | |
|  |  |
| Walking, MPA or VPA (h/day) | 107 965 (23%) |
|  |  |
| TV viewing | 4874 (1%) |
|  |  |
| BMI | 2626 (<1%) |
|  |  |
| Number of cancer and noncancer illnesses | 769 (<1%) |
|  |  |
| Townsend index | 597 (<1%) |
|  |  |
| Age at baseline | 3 (<1%) |
|  |  |
| **Categorical variables** | |
|  |  |
| Ethnicity | 2552 (<1%) |
|  |  |
| Alcohol intake frequency | 1369 (<1%) |
|  |  |
| Tobacco smoking frequency | 1198 (<1%) |
| MPA, moderate-intensity physical activity; VPA, vigorous-intensity physical activity; BMI, body mass index. | |

| **Supplementary Table 2.** Participant characteristics for those who did and did not die from COVID-19. | | |
| --- | --- | --- |
| Characteristic | No COVID-19 mortality  (N = 358 877) | COVID-19 mortality  (N = 879) |
| **Continuous variables** |  |  |
|  |  |  |
| Baseline age (years) | 56 (8) | 62 (6) |
|  |  |  |
| Current age (years)* | 67 (8) | 74 (6) |
|  |  |  |
| BMI (kg/m^2^) | 27.2 (4.6) | 29.8 (5.7) |
|  |  |  |
| Townsend Index^†^ | -1.5 (3.0) | -0.3 (3.5) |
|  |  |  |
| Number of cancer and noncancer illnesses | 2 (2) | 3 (2) |
|  |  |  |
| TV viewing (h/day) | 2.64 (1.62) | 3.45 (1.97) |
|  |  |  |
| Walking (h/day) | 0.76 (0.79) | 0.71 (0.75) |
|  |  |  |
| MPA (h/day) | 0.56 (0.73) | 0.59 (0.77) |
|  |  |  |
| VPA (h/day) | 0.21 (0.36) | 0.19 (0.38) |
|  |  |  |
| **Categorical variables** |  |  |
|  |  |  |
| ***Sex*** |  |  |
| Male | 168 827 (47%) | 596 (68%) |
| Female | 190 050 (53%) | 283 (32%) |
|  |  |  |
| ***Ethnicity*** |  |  |
| White | 341 352 (95%) | 812 (92%) |
| Non-white | 17 525 (5%) | 67 (8%) |
|  |  |  |
| ***Tobacco smoking frequency*** |  |  |
| On most or all days | 24 396 (7%) | 107 (12%) |
| Only occasionally | 9844 (3%) | 23 (3%) |
| Non-smoker | 324 637 (90%) | 749 (85%) |
|  |  |  |
| ***Alcohol intake frequency*** |  |  |
| Daily/almost daily | 76 516 (21%) | 177 (20%) |
| 1-4x/week | 180 720 (50%) | 365 (42%) |
| 1-3x/month | 39 642 (11%) | 85 (10%) |
| Special occasions/never | 61 999 (17%) | 252 (29%) |
|  |  |  |
| Characteristics presented as mean (standard deviation) for continuous variables, and number (percentage) for categorical variables.  *Age as of March 1^st^, 2020.  ^†^Higher values for Townsend Index imply a greater degree of deprivation.  BMI, body mass index; MPA, moderate-intensity physical activity; VPA, vigorous-intensity physical activity. | | |

| **Supplementary Table 3.** Sensitivity analysis - isotemporal substitution models examining the effect of replacing TV viewing with walking, MPA and VPA on COVID-19 mortality risk, with additional adjustment for BMI. | | | |
| --- | --- | --- | --- |
|  | Walking | MPA | VPA |
|  |  |  |  |
| Full sample | **0.87 (0.78 – 0.97)** | 0.95 (0.85 – 1.07) | 0.91 (0.74 – 1.12) |
|  |  |  |  |
| Males | 0.89 (0.78 – 1.01) | 1.00 (0.88 – 1.15) | 0.87 (0.69 – 1.11) |
|  |  |  |  |
| Females | 0.84 (0.69 – 1.01) | 0.85 (0.70 – 1.05) | 1.02 (0.68 – 1.55) |
|  |  |  |  |
| OR (95% CI) for COVID-19 mortality when replacing 1h of TV viewing with 1h of each physical activity.  All physical behaviours except for TV viewing were entered into the model with adjustment for total time in all behaviours (i.e., TV viewing + walking + MPA + VPA).  All models were adjusted for baseline age, sex (except when performing sex-specific analyses), BMI, ethnicity, tobacco smoking and alcohol intake frequency, Townsend Index, and number of cancer and noncancer illnesses.  BMI, body mass index; OR, odds ratio; CI, confidence interval; MPA, moderate-intensity physical activity; VPA, vigorous-intensity physical activity.  Significant associations (p<0.05) are highlighted in bold. | | | |
|  |  |  |  |
